# metaLINCS: an R package for meta-level analysis of LINCS L1000 drug signatures using stratified connectivity mapping

**DOI:** 10.1093/bioadv/vbac064

**Published:** 2022-09-09

**Authors:** Ivo Kwee, Axel Martinelli, Layal Abo Khayal, Murodzhon Akhmedov

**Affiliations:** BigOmics Analytics, 6500 Bellinzona, Switzerland; BigOmics Analytics, 6500 Bellinzona, Switzerland; BigOmics Analytics, 6500 Bellinzona, Switzerland; BigOmics Analytics, 6500 Bellinzona, Switzerland

## Abstract

**Summary:**

Accessing the collection of perturbed gene expression profiles, such as the LINCS L1000 connectivity map, is usually performed at the individual dataset level, followed by a summary performed by counting individual hits for each perturbagen. With the metaLINCS R package, we present an alternative approach that combines rank correlation and gene set enrichment analysis to identify meta-level enrichment at the perturbagen level and, in the case of drugs, at the mechanism of action level. This significantly simplifies the interpretation and highlights overarching themes in the data. We demonstrate the functionality of the package and compare its performance against those of three currently used approaches.

**Availability and implementation:**

metaLINCS is released under GPL3 license. Source code and documentation are freely available on GitHub (https://github.com/bigomics/metaLINCS).

**Supplementary information:**

Supplementary data are available at *Bioinformatics Advances* online.

## 1 Introduction

With the rapid proliferation of transcriptomic datasets, analysis has expanded beyond mere pairwise comparisons to identifying correlated perturbation signatures. In particular, the LINCS L1000 database (Duan *et al.*, 2014) has focused on detailing gene expression profiles based on the exposure of different cell lines to varying concentrations of various compounds, or following the alteration of the expression of a single gene.

It is often of interest to find drugs, or more generally ‘perturbagens’, which show similar profiles as some query signature. This is also known as ‘drug connectivity mapping’ (Lamb *et al.*, 2006). A typical use case for drug connectivity mapping (CMap) is represented by testing the transcriptomic activity of a novel drug and wanting to understand what other drugs showed similar expression profiles. This can be used to shed light into the mechanism of action (MoA) of the drug or to indicate possible target genes. Another typical use case for the drug CMap is the identification of drugs suitable for repurposing by looking at the differential expression between two conditions, for example diseased versus healthy, and identifying drugs that generate negatively correlated profiles.

These drug perturbation databases are generally large, with the L1000 databases consisting of over 1 million profiles. A particular complication of the L1000 database is that only an incomplete subset of genes (about 1000) has been measured. Another complication is that drugs are typically tested numerous times on different cell lines and at different concentrations.

Our package, which we named metaLINCS, proposes a novel approach to correlate LINCS L1000 perturbation signatures. Rather than providing outputs for each perturbation experiment individually, we take the output and perform a meta-level gene set enrichment analysis (GSEA) (Subramanian *et al.*, 2005) by combining them by compound, based on the ranked correlation scores for each experiment. Finally, we perform an additional meta-level GSEA computation step to combine compounds into MoAs or target genes, to highlight overarching themes in the data.

Several tools provide analysis based on the L1000 database such as enrichR (Chen *et al.*, 2013), iLINCS (Pilarczyk *et al.*, 2020) and more recently, SigCom LINCS (Evangelista *et al.*, 2022). Table 1 compares the features of our method with the aforementioned methods. Note that only metaLINCS provides meta-level enrichment analysis beyond single experiments.

**Table 1.** Feature comparison between metaLINCS and other drug connectivity methods

	metaLINCS	EnrichR	iLINCS	SigCom
L1000 by experiment		✓	✓	✓
L1000 by compound	✓			
MoA enrichment	✓			
Target gene enrichment	✓			
Pathway enrichment^a^		✓		

*Note:* Only our method provides meta-level enrichment analysis for compound, MoA and target gene.

a Generic pathway enrichment such as GO, KEGG or MSigDB.

Although metaLINCS was intended for the LINCS L1000 database, the method can be used for general perturbation enrichment analysis, where repetition of experiments under similar, but not identical, conditions (e.g. different drug concentrations or different cell lines) is a concern. Such collections include various drug sensitivity databases, such as the Genomics for Drug Sensitivity in Cancer (GDSC) database (Yang *et al.*, 2013) or the Cancer Therapeutics Response Portal (CTRP) (Basu *et al.*, 2013). metaLINCS is available on GitHub and is also available in Omics Playground, a platform we developed previously (Akhmedov *et al.*, 2020).

## 2 Methods

**Fig. 1.**
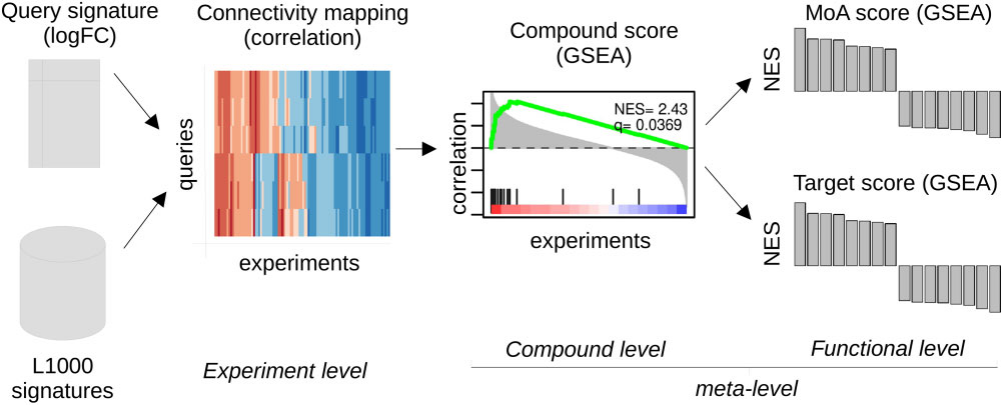
Algorithm flowchart of metaLINCS: the analysis starts by computing experiment connectivities from a query signature and L1000 signatures. An initial GSEA step computes the normalized enrichment score (NES) value as a compound score from the previously computed connectivity values. This is followed by a second GSEA step that computes the NES value as a MoA/target score from the obtained compound scores

Figure 1 depicts the algorithm flowchart of our method. metaLINCS needs a real-valued fold-change vector (or matrix for multiple contrasts) as query input, a reference perturbation database and an MoA annotation table. Here, we are using the L1000 database and corresponding annotation tables.

The analysis starts by calculating the rank correlation, or ‘connectivities’, between the gene expression levels of the test profile *y* with each given perturbagen profile *x_i_* for experiment *i* in the perturbation database:

(1) $$\rho_{i}=\text{cor}(R(x_{i}),R(y))$$

where *ρ_i_* is a scalar value and *R* is the rank transform operator.

We define a perturbagen set $D_{j}$ associated with a perturbagen *d_j_* as:

(2) $$p_{j}=\{i\;\;|\;\;\text{perturbagen}(i)=d_{j}\}$$

where *i* is the index over all experiments, so that set $D_{j}$ contains all experiment IDs that are associated with perturbagen *d_j_*. We can then perform a GSEA across all the experiments for a given perturbagen *d_j_*, by using *D_j_* and the previously calculated correlation coefficients *ρ* as ranking metrics. From the GSEA, we can obtain the ’perturbagen score’ *x_j_* for perturbation *d_j_* as the normalized enrichment score (NES)

(3) $$x_{j}=\text{NES}(D_{j},\rho ).$$

**Fig. 2.**
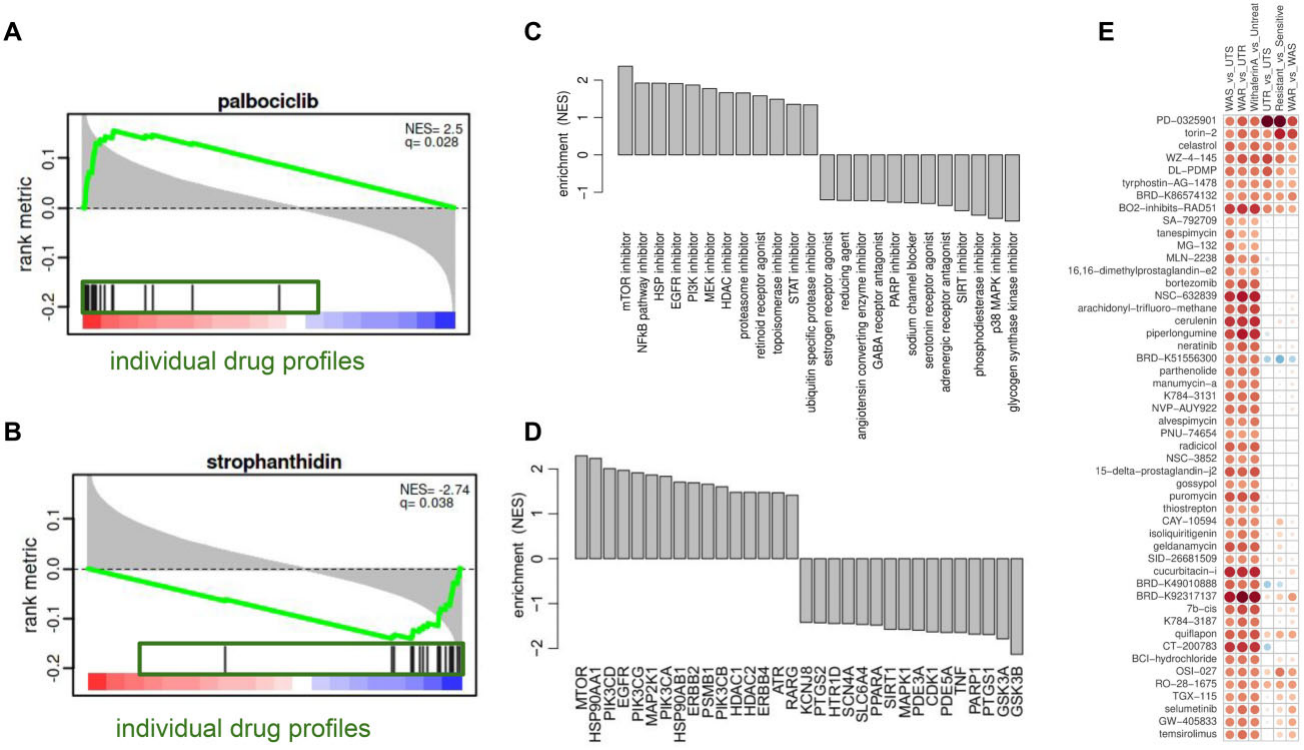
Visual outputs of the package. (**A** and **B**) Two GSEA plots showing a positively (**A**) and negatively (**B**) correlated profile based on the correlation scores of individual drug profiles (green boxes) with a test profile; individual black bars denote ranks based on the correlation coefficients for all experiments corresponding to a particular drug (here palbociclib and strophanthidin). The package correlates a test signature with known drug profiles from the L1000 database and shows similar or opposite profiles by running the GSEA algorithm on the drug profile correlation space. (**C**) Barplot displaying the most enriched compound MoAs. On the vertical axis, the GSEA normalized enrichment score of the MoA class is plotted. (**D**) Barplot displaying the most enriched target genes. On the vertical axis, the GSEA normalized enrichment score of the gene target is plotted. (**E**) An activation matrix showing the most positively or negatively correlated perturbagens across all test profiles. The size of the circles correspond to their relative activation, and are colored according to their upregulation (red) or downregulation (blue) in the contrast profile

Example enrichment plots for palbociclib and strophanthidin are shown in Figure 2A and B for a positively and negatively correlated enrichment, respectively. Notice that in this case, instead of showing ranked genes, the plot shows the sorted correlation for each individual experiment. The NES indicates the presence of a positive or negative correlation between the cumulative list of experiments for a given perturbagen and the test profile, while a *q*-value indicates statistical significance. For increased statistical robustness, we recommend the exclusion of any perturbagens with <20 experiments in the database. For compounds only, metaLINCS repeats an additional GSEA analysis step to identify those MoA with a positive or negative correlation with the test profile by grouping the various drugs with the same MoA. We define a ‘MoA set’ $M_{j}$ associated with MoA *m_j_* as:

(4) $$M_{j}=\{i\;\;|\;\;\text{MoA}(i)=m_{j}\}$$

where *i* is the index over all perturbagens, so that set $M_{j}$ contains all perturbagen IDs that are associated with MoA *m_j_*. Then GSEA is performed using all $M_{j}$ and the previously computed ‘perturbagen enrichment’ score *p*. We can obtain the ‘MoA score’ *q_j_* for each MoA class *m_j_* as

(5) $$q_{j}=\text{NES}(M_{j},p).$$

where NES is the normalized enrichment score from the GSEA analysis.

Similarly, we can define a ‘Target set’ $T_{j}$ associated with target *t_j_* as:

(6) $$T_{j}=\{i\;\;|\;\;\text{target}(i)=t_{j}\}$$

where *i* is the index over all perturbagens, so that set $T_{j}$ contains all perturbagen IDs that are associated with target *t_j_*. Then GSEA is performed using all $T_{j}$ and the previously computed ‘perturbagen enrichment’ score *p*. We can obtain the ‘target score’ *r_j_* for each target class *t_j_* as

(7) $$r_{j}=\text{NES}(T_{j},p).$$

The package then displays the MoA scores *q* as a barplot sorted by highest and lowest scores respectively (Fig. 2C and D). Using a target scores *r*, the plot can also be set to display the most enriched target genes, instead of the most enriched MoA class. In the case of multiple test profiles, the package also generates an activation matrix that highlights the most positively or negatively correlated perturbagens across all available test profiles (Fig. 2E). Finally, a complete table of each drug with their raw and adjusted *P*-values, MoA and target genes is produced. Using this approach, positively or negatively correlated perturbagens can be identified more robustly and reliably by combining the results from the different experiments, rather than considering them individually. However, as a drawback, correlations with under-represented perturbagens (in this case 20 experiments) will be missed.

Instead of the L1000 perturbation dataset, metaLINCS can be used with other reference datasets, for example, instead of the L1000 perturbation database that measure ‘drug activity’, one could prepare reference sets from the GDSC or CTRP databases that measure ‘drug sensitivity’ instead. For this, the user needs to appropriately prefix the profile IDs in the header separated by an ‘underscore’ or ‘at’ symbol: *drug*_ *profileID* or *drug*@*profileID*. This will group all profiles with the same prefix to the same corresponding perturbagen/drug. For MoA and gene target meta-level analysis, the method needs an additional MoA annotation file with at least the columns ‘drug’, ‘moa’ and ‘target’. More details are provided in the source code on GitHub.

## 3 Results

We tested our package on a publicly available dataset that was used to test the efficacy of Withaferin A, a natural product with a wide range of pharmacological activities, against glucocorticoid (GC)-sensitive and -resistant Multiple Myeloma cells (Logie *et al.*, 2021). Comparing treated versus untreated sensitive samples indicated that Withaferin A shared MoAs with heat shock protein (HSP), mTOR, PI3K, NF-*κ*B pathway, histone deacetylase, MEK, proteasome, topoisomerase and epidermal growth factor receptors inhibitors among the most correlated matches from the L1000 database (Fig. 2C). This was consistent with the various pharmacological activities attributed to Withaferin A in the literature (Bungau *et al.*, 2021; Dom *et al.*, 2020; Malik *et al.*, 2021) and to the related Withangulatin A compound (Lee *et al.*, 1991). Comparing untreated GC-resistant versus untreated GC-sensitive samples highlighted three compounds with unknown MoA and strophanthidin (Fig. 2B) as having the lowest NES in the list. A negative NES indicates that a compound could counteract a given gene expression profile. In the case of strophanthidin, evidence does indeed support its role as a drug that could counteract the GC-resistant phenotype: it is an inhibitor of the MAPK signaling pathway (Reddy *et al.*, 2019) and MAPK signaling pathway inhibition was shown to reverse GC resistance in leukemia (Jones *et al.*, 2015).

To assess the performance of metaLINCS against other publicly available approaches, we selected five recent public datasets from the GEO database: GSE165325, GSE137535, GSE182018, GSE193402 and GSE192446. These datasets contained comparisons between samples treated with five different drugs (namely bortezomib, dexamethasone, homoharringtonine/omacetaxine mepesuccinate, sirolumus/rapamycin and vorinostat, respectively) against untreated controls and were selected based on the presence of corresponding profiles in the LINCS1000 database and to capture different MoAs. Specifically, we used the 1 μM rapamycin experiment from dataset GSE193402 and the vorinostat-treated JEG-3 cell line from dataset GSE192446. We then used three commonly used approaches (the aforementioned EnrichR, iLINCS and SigCom) on a list of up- and downregulated genes (FDR < 0.05 and a |logFC|>0.5) for each dataset obtained by intersecting the results of DESeq2 (Love *et al.*, 2014), EdgeR (Robinson *et al.*, 2010) and limma (Ritchie *et al.*, 2015). It should be noted that it is not straightforward to compare the methods, as metaLINCS produces a combined score for all the experiments of a given chemical perturbagen, while the other approaches provide a score for each experiment individually, although iLINCS also provides the cumulative number of enriched signatures per chemical perturbagen. Furthermore, as previously mentioned, metaLINCS also provides a score by MoA, while only iLINCS produces something comparable by ranking the total number of enriched signatures by target genes.

To compare the relative performance of the methods, we computed the drug signatures for each test data. For EnrichR and SigCom, we ran separate lists for up- and downregulated genes. We then ranked the first occurrence of a compound with a related MoA in each of the lists produced by metaLINCS, EnrichR, SigCom and iLINCS (Table 2). When comparing the ranking results using this heuristic, metaLINCS outperformed the other methods, although it should be added that iLINCS performance improves when also considering cumulative enriched signatures by compound (e.g. bortezomib is the most common chemical perturbagen among enriched signatures for its corresponding dataset).

**Table 2.** Comparison of L1000 connectivity methods including metaLINCS

	metaLINCS	EnrichR	SigCom	iLINCS
Bortezomib^a^	1st	>100th	>100th	17th
Dexamethasone^b^	2nd	>100th	2nd	2nd
Homoharringtonine^c^	1st	>100th	4th	3rd
Sirolimus^d^	1st	1st	3rd	2nd
Vorinostat^e^	1st	1st	1st	2nd

*Note*: Ranks of the first occurrence of a compound with a related MoA on five public GEO datasets.

a GSE165325,

b GSE137535,

c GSE182018,

d GSE193402 and

e GSE192446.

As mentioned above, only metaLINCS and iLINCS produce an output from which the MoA of a given drug can be extrapolated. metaLINCS correctly identified the MoA for four of the compounds, with the sole exception being bortezomib, where the main MoA was indicated as ‘HSP inhibitor’ and the correct label ‘proteasome inhibitor’ only appeared fourth in the list (Supplementary Fig. S1). However, metaLINCS correctly identified bortezomib as the most positively correlated drug (Supplementary Fig. S2). Conversely, iLINCS correctly identified the main MoA, as extrapolated from the cumulative target genes ranking, for three of the drugs. No target genes were associated with homoharringtonine, while the main target gene associated with dexamethasone was indicated as MAP2K1, rather than the GC receptor NR3CI, which ranked fourth. Nonetheless, three of the top five experimental drug profiles identified in iLINCS correctly consisted of GC receptor agonists.

Overall, the performance of metaLINCS is at least comparable (if not slightly superior) to that of iLINCS, the best performing of the three current approaches considered for evaluation.

## 4 Conclusion

The metaLINCS package provides a novel approach to evaluate the correlation between test gene expression profiles and perturbation signatures from the L1000 database. It simplifies the results of a rather complex analysis by performing additional meta-level enrichment steps and provides a robust and reliable identification of perturbagens and MoAs with positively or negatively correlated expression profiles to a given test profile.

## Author contributions

I.K. and M.A. conceptualized the method. A.M., L.A.L. and I.K. wrote the manuscript. I.K. did initial coding. L.A.L. created the R package.

## Funding

None declared.

*Conflict of Interest*: I.K., A.M. and M.A. are employees of BigOmics; L.A.L. was on an internship at BigOmics.

## Supplementary Material

vbac064_Supplementary_Data
