# Supplementary figures and images for: metaLINCS: an R package for meta-level analysis of LINCS L1000 drug signatures using stratified connectivity mapping
Source: Bioinform Adv. 2022 Sep 9;2(1):vbac064. doi: 10.1093/bioadv/vbac064 (PMC9710587; doi:10.1093/bioadv/vbac064)

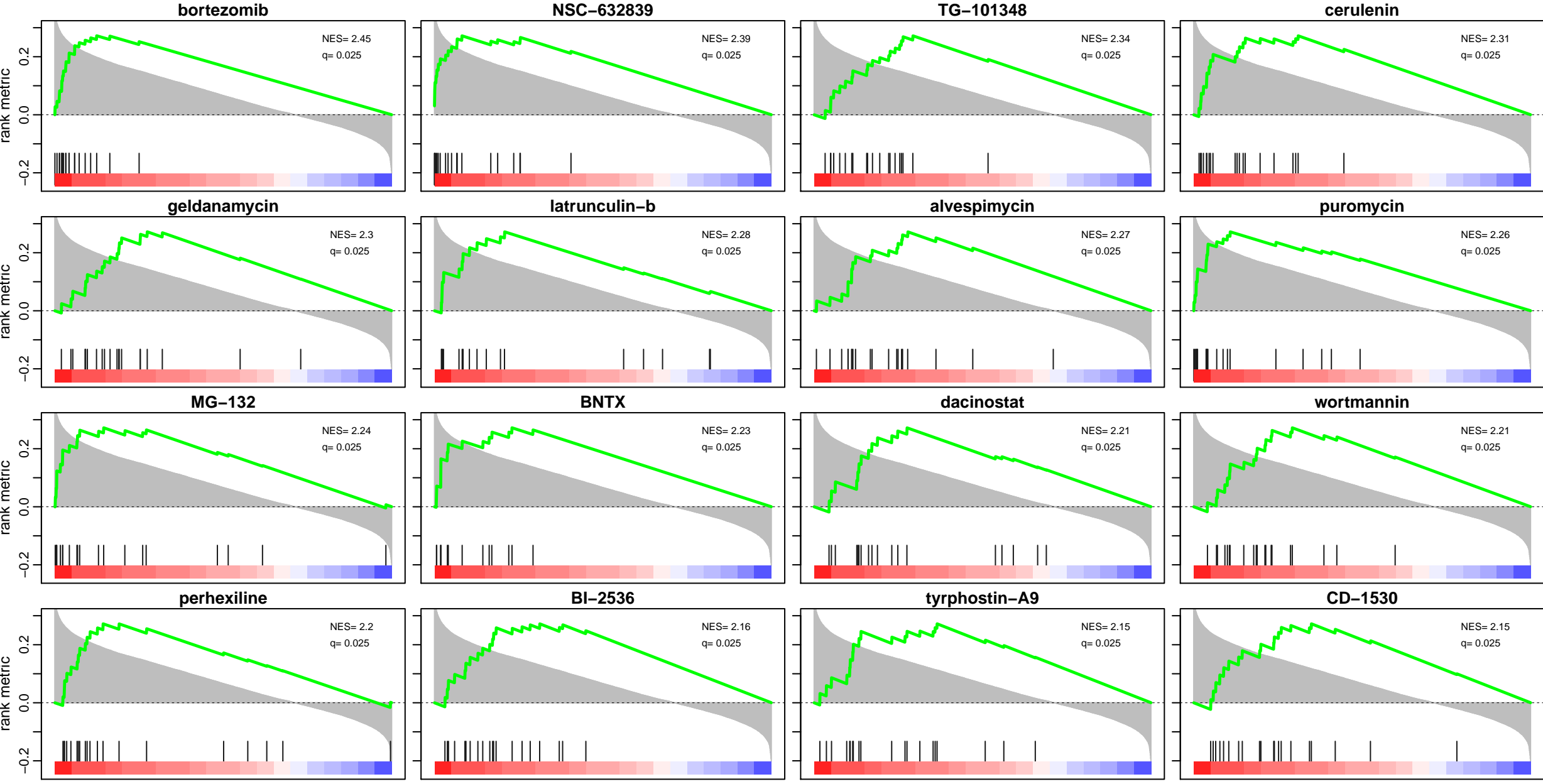

Supplement: vbac064_Supplementary_Data [file vbac064_supplementary_data.zip › Supplementary_figure_2.pdf]

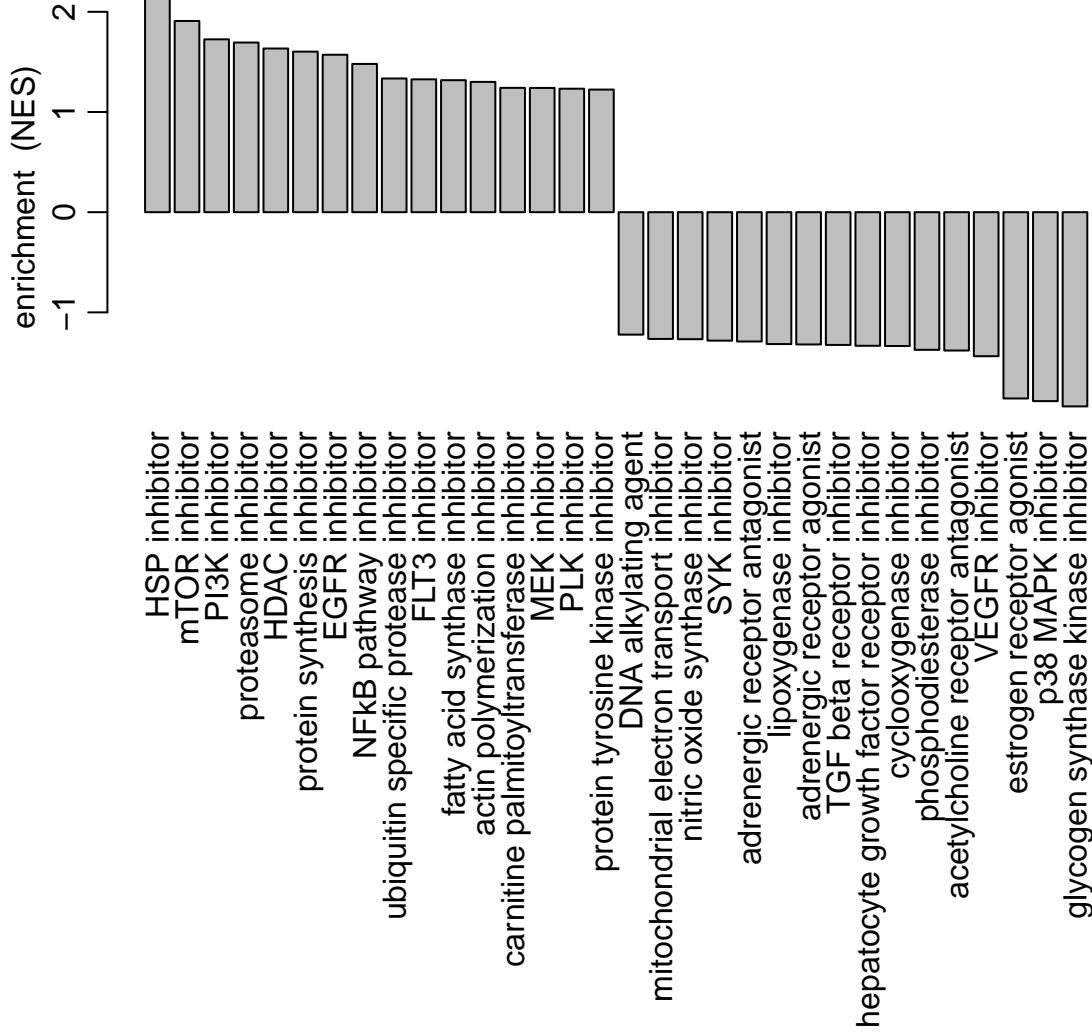

Supplement: vbac064_Supplementary_Data [file vbac064_supplementary_data.zip › Supplementary_figure_1.pdf]
